# 25-Hydroxyvitamin D in Cancer Patients Admitted to Palliative Care: A Post-Hoc Analysis of the Swedish Trial ‘Palliative-D’

**DOI:** 10.3390/nu14030602

**Published:** 2022-01-29

**Authors:** Maria Helde Frankling, Caritha Klasson, Linda Björkhem-Bergman

**Affiliations:** 1 Karolinska Institutet, Department of Neurobiology, Care Sciences and Society (NVS), Division of Clinical Geriatrics, Blickagången 16, Neo Floor 7, SE-141 83 Huddinge, Sweden; caritha.klasson@ki.se (C.K.); linda.bjorkhem-bergman@ki.se (L.B.-B.); 2 Thoracic Oncology Center, Department of Oncology-Pathology, Karolinska Institutet, Karolinska University Hospital, SE-171 76 Stockholm, Sweden; 3 Stockholms Sjukhem, Palliative Medicine, Mariebergsgatan 22, SE-112 19 Stockholm, Sweden

**Keywords:** vitamin D, cholecalciferol, 25-OHD, vitamin D deficiency, palliative, cancer, latitude, tumor type, season, sex differences

## Abstract

The purpose of this study is to explore 25-hydroxyvitamin D (25-OHD) levels in patients with cancer in the palliative phase in relation to season, sex, age, tumor type, colectomy, and survival. To this end, we performed a post-hoc analysis of ‘Palliative-D’, a randomized placebo-controlled, double-blind trial investigating the effect of daily supplementation with 4000 IU of vitamin D for 12 weeks on pain in patients in palliative cancer care. In the screening cohort (*n* = 530), 10% of patients had 25-OHD levels < 25 nmol/L, 50% < 50, and 84% < 75 nmol/L. Baseline 25-OHD did not differ between seasons or tumor type and was not correlated with survival time. In vitamin D deficient patients supplemented with vitamin D (*n* = 67), 86% reached sufficient levels, i.e., >50 nmol/L, after 12 weeks. An increase in 25-OHD was larger in supplemented women than in men (53 vs. 37 nmol/L, *p* = 0.02) and was not affected by season. In the placebo-group (*n* = 83), decreased levels of 25-OHD levels were noted during the study period for patients recruited during the last quarter of the year. In conclusion, cancer patients in palliative phase have adequate increase in 25-OHD after vitamin D supplementation regardless of season, age, tumor type, or colectomy.

## 1. Introduction

Vitamin D is a hormone mainly synthesized in the skin in the presence of sunlight, with 7-deoxycholesterol as a substrate [1]. Smaller amounts of vitamin D are ingested orally, through foodstuffs and supplementation products [2]. Vitamin D is activated in two hydroxylation steps into the active form, 1,25-dihydroxyvitamin D [1]. The active form of vitamin D is the only known ligand to the vitamin D receptor (VDR), a nuclear receptor present in many different cell types [3]. Vitamin D plays an important role in maintaining calcium homeostasis [1], in skeletal health [1], and in the immune system [4,5]. The individual’s vitamin D levels is assessed by 25-hydroxyvitamin D (25-OHD), a more stable compound than 1,25(OH)2D [6]. 25-OHD levels below 25 nmol/L constitute severe deficiency and between 25 and 50 nmol/L deficiency [7]. Levels above 50 nmol/L are considered to ensure skeletal health, while 75 nmol/L may be needed for optimal functioning of the immune system [7]. Toxic levels that can cause hypercalcemia and renal failure are identified as levels above 250 nmol/L [7]. Cross sectional data on 25-OHD levels and mortality do however suggest a U-shaped relationship, where levels above 125 nmol/L are not necessarily beneficial for the individual [8].

Mechanistic studies suggesting anti-proliferative and anti-inflammatory effects of vitamin D [4,5,9,10], have spiked interest in epidemiological vitamin D research in cancer patients. Studies on cancer incidence and mortality indicate that vitamin D supplementation may reduce cancer specific, but not overall, mortality [11,12,13,14,15]. Prospective clinical studies investigate the possible potentiating effect of vitamin D on oncologic treatment effect [16,17], as well as its possible role in the management of pain [18,19,20,21,22], fatigue, and quality of life [20,21,23,24,25]. In a recent US study, 56% of cancer survivors took vitamin D supplementation compared to 37% in the general population [26]. This is a large increase in numbers compared with older cohorts, where fewer than 20% of cancer patients took vitamin D [27].

In Sweden, synthesis of vitamin D cannot take place between October and March (“vitamin D-winter”) [28]. Foods are fortified with vitamin D, and risk groups are recommended supplementary vitamin D intake [2]. Still, there is a significant seasonal variation in vitamin D levels [29,30,31], and 50% of healthy Swedish adults have vitamin D levels below 50 nmol/L during winter [29,31,32]. Although oral vitamin D intake has increased over time [33], vitamin D levels have remained constant [32]. In institutionalized patients in Swedish care homes, most patients were vitamin D deficient [34,35]. In contrast, community-dwelling elderly Swedes have much higher vitamin D levels [36,37,38,39]. In Supplementary Materials Table S1, we present cross sectional Swedish studies on 25-OHD levels.

In cross sectional studies on 25-OHD levels in advanced or metastatic cancer patients, 25-OHD levels differ greatly between cohorts [40,41,42,43,44,45,46], with no seasonal variation in an Australian cohort [45]. In Table 1, we present an overview of cross-sectional data from cohorts of patients with palliative stage cancer disease.

In the randomized, placebo-controlled, double-blind trial ‘Palliative-D’, we investigated the effect of 12 weeks of supplementation with 4000 IU vitamin D3 to patients with advanced or metastatic cancer and 25-OHD ≤ 50 nmol/L on pain, infections, fatigue, and quality of life (QoL) [20,47]. The mean change in opioid dose (as a proxy for pain) was lower in vitamin D supplemented patients than in controls. Vitamin D treated patients were also less fatigued. There was no difference between groups regarding antibiotic use (as a proxy for infections), or QoL [47].

We have identified a knowledge gap regarding 25-OHD levels in palliative cohorts from Northern latitudes, as well as the effect of vitamin D supplementation in palliative care cohorts with mixed cancer types. In this post-hoc analysis of the randomized, controlled trial (RCT) Palliative-D’, the primary aim is to explore 25-OHD levels in relation to season, age, and tumor type in patients with advanced cancer, as well as change in 25-OHD in both untreated and vitamin D supplemented patients. We hypothesize that this severely diseased cohort presents smaller seasonal variations compared to healthier Swedish cohorts due to more time spent indoors and thus experiencing less sun exposure during summer months.

**Table 1:** Cross-sectional cohorts with measurements of 25-OHD in patients with mixed tumor types in a palliative setting.

Author, Year	Cohort, Location Year	Study Population, Sex, Age	25-OHD, nmol/L Median(Min-Max) or Mean (SD)	Seasonal Variation in 25-OHD	Proportions of Vitamin D Deficient Participants 25-OHD, nmol/L	Vitamin D Supplementation/ Other Comment
Alkan 2019 [40]	Outpatient cancer clinic, Turkey December 2016–May 2018	*n* = 706 41% men	30.5 (5–241)	Summer: 67% < 50 Autumn: 65% < 50 Winter: 77% < 50 Spring: 78% < 50	Palliative: 76% < 50	Vitamin D supplementation = exclusion criterion
Dev 2011 [42]	Cancer patients, supportive care clinic US 2009–2010	*n* = 100 68% men median age 60	No information	No information	47% < 50 70% < 75	Deficiency more common in non-whites and females. 15–19% on vitamin D supplementation
Morton 2014 [45]	Oncology/palliative care unit, 76% metastatic disease Australia, 27° S	*n* = 100	Mean 54.6	Higher mean in spring, but only 4 observations	44% < 50 16% < 30 1% < 12.5	No supplements. No association 25-OHD-cancer type. PS associated with 25-OHD.
To 2011 [46]	Inpatient hospice, Australia	*n* = 21, 52% men mean age 69	41 (17–100) 47.5 (23.4)	All measurements during summer	72% < 60	
Edwards 2018 [43]	Cancer patients Texas, US, 2013–2015		No information	No information	49% < 75	No information on metastatic disease
Martinez-Alonso 2016 [44]	Cancer patients (palliative), Spain, March 2013–Aug 2014	*n* = 30, 77% men mean age 63	No information	No information	90% < 75 40% < 20	No supplements. PS and fatigue correlated with 25-OHD
Bergman 2015 [41]	Palliative care Unit Stockholm, 59° N, April 2014–January 2015	*n* = 100 43% men median age 71	40 (8–154)	No information	65% < 50	Lower 25-OHD in patients who died during follow-up (36 vs. 50, *p* = 0.013)
Wang-Gillam 2008 [48]	Breast cancer, Arkansas, US, 2002–2006	*n* = 21 100% women	No information	No information	48% < 50 67% < 75	Patients with metastatic disease in a larger cohort
Solomon 2012 [49]	Advanced malignancy and pain Connecticut, US	*n* = 260	No information	No information	21% < 25 43% 25–50 20% 50–75 8% > 75	Poster abstract, no detailed information on supplementation

Abbreviations: 25-OHD: 25-hydroxyvitamin D, °N: degrees North (latitude), PS: Performance Status, °S: degrees South (latitude), SD: Standard deviation, US: United States.

## 2. Materials and Methods

Patients were all in a palliative phase of their disease trajectory and they were recruited from advanced palliative home care teams in the Stockholm Region (59° N) between November 2017 and March 2020. Vitamin D levels (25-OHD) were assessed as part of the screening procedure in all consenting patients (*n* = 530), fulfilling inclusion and exclusion criteria. At screening, information on age, sex, and type of cancer was retrieved from medical records [20]. The original study did not comprise assessment of food intake or more specifically vitamin D ingestion.

**Figure 1:**
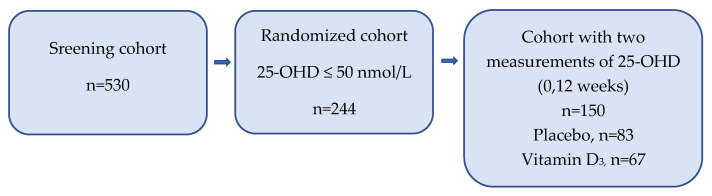
‘Palliative-D’ cohorts analyzed regarding 25-OHD levels.

Patients with 25-OHD ≤50 nmol/L (*n* = 244) were randomized to study drug, *n* = 121 to vitamin D3 oil drops (Detremin) 4000 IU/day and *n* = 123 to placebo [47]. Patients completing all 12 weeks of intervention (*n* = 150) had their 25-OHD levels measured again at the end of the study (Figure 1). Only 61% of randomized patients could be evaluated after twelve weeks, with clinical deterioration and death due to malignancy causing high attrition rates. In the results section, we present data on the screening cohort (*n* = 530) and the randomized cohort with two measurements of 25-OHD with a 12-week interval (*n* = 150) under different subheadings (Figure 1).

Some data on 25-OHD levels have been presented in previous publications on the studied cohort. In the screening cohort, median 25-OHD was 51 nmol/L (range 8–195) in both men and women [50]. Median baseline values of 25-OHD in randomized patients was 38 nmol/L (IQR 28–45) [47]. In the placebo group, median 25-OHD remained unchanged. In patients supplemented with vitamin D, 25-OHD increased from 36 (±11) to 81 (±26) nmol/L (*p* < 0.001) [47].

As previously reported, the median age in the entire screening cohort was 70 years (IQR 62–77) [50], and the median age was 68 years (IQR 61—75) in patients randomized to study the drug [47]. There were equal numbers of men and women in the screening cohort, with 265 in each group [50]. In randomized patients with two assessments of 25-OHD (*n* = 150), 49% were men [47]. In both the screening and in the randomized cohorts, colorectal cancer was the most common tumor type, followed by upper gastrointestinal (GI) and lung cancer [47,50]. We did not collect data on physical performance status or socioeconomic factors.

Inclusion criteria allowed for a daily dose of 400 IU vitamin D, and patients were meticulously asked about nutritional supplements during the screening process, so as to avoid recruiting patients who were taking larger than allowed doses of vitamin D [47]. We only recruited patients who planned to spend the next 12 weeks in the Stockholm region, but are aware that a few recruited participants still went on shorter holiday trips during winter months. Compliance was overall good, however 2 patients in the intervention group reported lacking compliance. Compliance is reported in greater detail in the supplementary material of the original publication [47].

Vitamin D levels were assessed as 25-OHD in serum analyzed by chemiluminescence immunoassay (CLIA) on a LIAISON-instrument (DiaSorin Inc, Stillwater, MN, USA) with a detectable range of 7.5 ± 175 nmol/L, CV 2 ± 5% at the Department of Clinical Chemistry, Karolinska University Hospital.

Statistical analysis was performed using Graph-Pad Prism version 8.4.3. Data that do not show Gaussian distribution, medians, IQR, and min-max are presented. For data with Gaussian distribution, we also calculated means and standard deviations (SD). Two tailed significance tests with a significance level of 0.05 were performed with Mann–Whitney U for non-normally distributed data and with Fisher’s exact test for normally distributed data. Baseline 25-OHD in relation to tumor type and change in 25-OHD in relation to tumor type in non-supplemented patients were compared using the Kruskal Wallis test. Proportions of categorical variables were compared with Fisher’s exact test. An analysis of correlation between 25-OHD and survival was done with simple linear regression.

## 3. Results

### 3.1. Baseline Characteristics Not Reported in Previous Publications

In randomized patients, 25-OHD levels ranged from 8–50 nmol/L. Vitamin D levels at screening in patients randomized to intervention were lower in patients who did not complete all 12 weeks, compared to those who did (median 25-OHD 34 vs. 39 nmol/L), however the difference was not statistically significant (*p* = 0.075).

### 3.2. Cutoff Levels for Vitamin D Deficiency

#### 3.2.1. Screening Cohort

In the screening cohort, 10% of patients had 25-OHD levels < 25 nmol/L, 50% < 50 nmol/L, and 84% < 75 nmol/L. Two percent of screened patients had 25-OHD above 125, with the highest individual value at 195 nmol/L. Two patients in the screening cohort had undetectable vitamin D levels at screening (<8 nmol/L), one of them being a patient with breast cancer who died shortly after inclusion. The other patient with unmeasurable 25-OHD also had a diagnosis of breast cancer, and was randomized to vitamin D supplementation 4000 IU/day in September and increased 25-OHD to 130 nmol/L after 12 weeks of follow up (the largest individual increase in the intervention group).

#### 3.2.2. Randomized Cohort

In patients who received vitamin D supplementation for 12 weeks (*n* = 67), 13% had baseline values < 25 nmol/L and 15/67 patients with initial values below 50 nmol/L reached levels above 100 nmol/L after 12 weeks (median increase in this subpopulation was 79 nmol/L, IQR 64–94). In contrast, 9/67 vitamin D supplemented patients remained vitamin D deficient. In this group, 8/9 patients were male and 7/9 had gastrointestinal tumors. One patient with a gastrointestinal neuroendocrine tumor (GI-NET) and one with pancreatic malignancy dropped their 25-OHD-levels with two units during follow-up. Very small increases in 25-OHD (2–5 nmol/L) were seen in three colorectal cancer patients, two of which had undergone total or partial colectomy. Still, median 25-OHD levels in all patients with gastrointestinal cancer were not significantly lower than other tumor groups. Change in 25-OHD in relation to cutoff values for vitamin D deficiency are presented in Supplementary Materials Table S2.

### 3.3. 25-OHD in Relation to Season

#### 3.3.1. Screening Cohort (*n* = 530)

**Figure 2:**
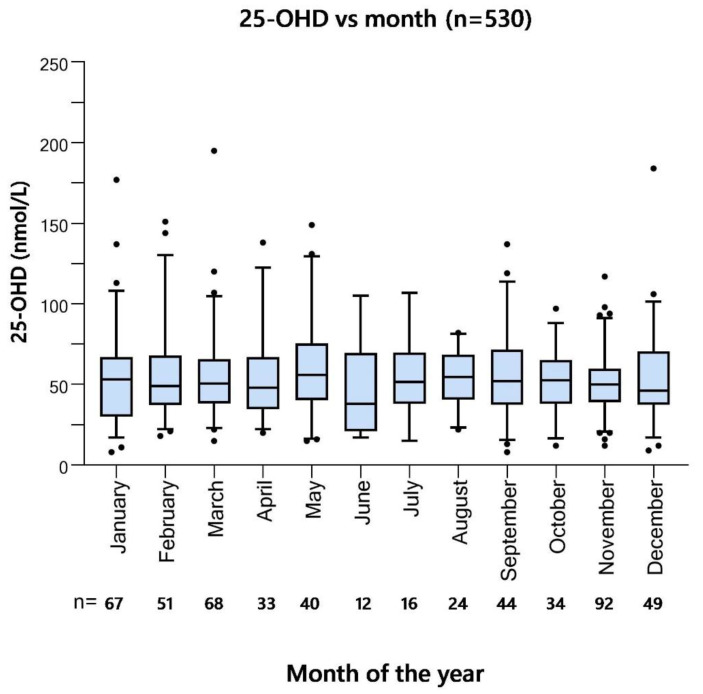
Cross-sectional 25-OHD in relation to screening month. Median 25-OHD values in nmol/L in the screening cohort (*n* = 530) of the ‘Palliative-D’ study. Boxes show interquartile range whiskers 5/95 percentiles and dots outliers. There were no statistically significant differences between groups (Mann-Whitney U).

Baseline 25-OHD did not differ significantly between months or quarters of the year or summer (April–September) versus winter season (October–March) in the screening cohort (Figure 2, Supplementary Materials Table S3). There were differences in the number of patients screened each month, with the lowest numbers in June (*n* = 12) and July (*n* = 16), and the highest numbers in November (*n* = 92), March (*n* = 68), and January (*n* = 67). Only 10 percent of patients were recruited during the summer months June, July, and August (52/530).

#### 3.3.2. Randomized Cohort (*n* = 150)

In patients receiving placebo, the difference in median change in 25-OHD over 12 weeks for patients recruited during the first quarter of the year (3 nmol/L) was significantly higher than in those recruited during Q4 (−3 nmol/L *p* = 0.003) (Supplementary Materials Table S4). In patients supplemented with 4000 IU vitamin D/day, median change in 25-OHD for patients recruited during the first two quarters of the year was 44 and 47 nmol/L respectively, and 36.5 and 37 nmol/L in Q3 and Q4, however differences between time periods were not significant (Supplementary Materials Table S4).

### 3.4. Change in 25-OHD in Relation to Sex, Randomized Cohort (n = 150)

In patients receiving placebo, there was no difference in median change in 25-OHD over 12 weeks between men and women (data not shown). In patients supplemented with 4000 IU vitamin D for 12 weeks (*n* = 67), a median increase in 25-OHD for men was 37 nmol/L and for women, it was 53 nmol/L (confidence interval, CI, for difference between groups −26 to −2, *p* = 0.02). The difference between groups was mainly due to large increases in 25-OHD in a small number of women, i.e., outliers.

### 3.5. 25-OHD in Relation to Cancer Type

#### 3.5.1. Screening Cohort (*n* = 530)

**Figure 3:**
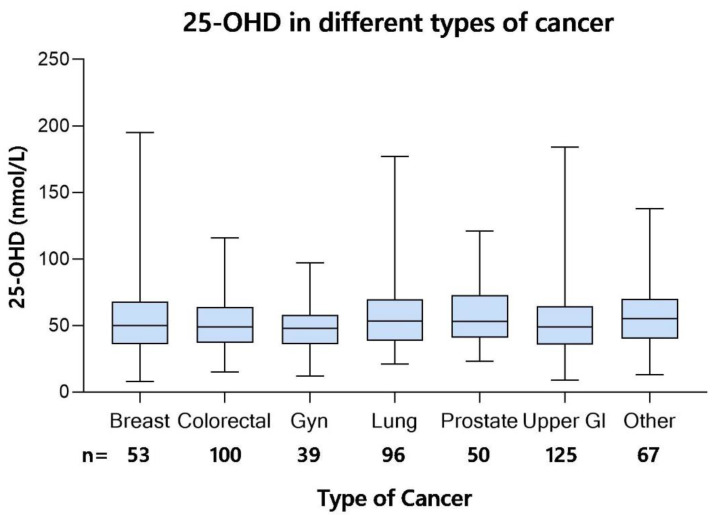
Cross-sectional 25-OHD in relation to tumor type (*n* = 530). Median 25-hydroxyvitamin D (25-OHD) values in nmol/L in patients with different types of cancer from the screening cohort of the ‘Palliative-D’-study. Boxes show interquartile range and whiskers min-max values. There were no statistically significant differences between groups (Kruskal Wallis).

In the screening cohort, there were no significant differences in 25-OHD between patients with breast, colorectal, lung, gynecological, prostate cancer, upper gastrointestinal (GI) cancer, or “other”, a group in which tumor types with fewer observations were pooled (cancer of unknown primary, tumors of the central nervous system, head & neck cancer, hematological malignancy, malignant melanoma, sarcoma, and urinary tract tumors) (Figure 3).

#### 3.5.2. Randomized Cohort (*n* = 150)

A change in 25-OHD over time in patients receiving placebo did not vary between cancer types (*p* = 0.56). In vitamin D supplemented patients, the median change in 25-OHD was largest in patients with gynecological tumors (*n* = 7, median 64, IQR 54–86) and lowest in patients with prostate cancer (*n* = 7, median 26, IQR 21–58). Due to few observations, we did not perform a significance test for change in 25-OHD in supplemented patients across all tumor types. When comparing median change in 25-OHD between the two largest supplemented groups, colorectal cancer and upper GI-cancer, results were very similar (data not shown). When looking at individual values, all vitamin D supplemented patients with very small changes in 25-OHD after 12 weeks had GI-tumors.

Median 25-OHD values in nmol/L in the screening cohort (*n* = 530) of the ‘Palliative-D’ study included the interquartile range (boxes) and min-max values (whiskers). Comparisons between groups was performed with Mann–Whitney U, and no significant difference between the types of cancer was observed.

### 3.6. 25-OHD in Relation to Age, Screening Cohort (n = 530)

**Figure 4:**
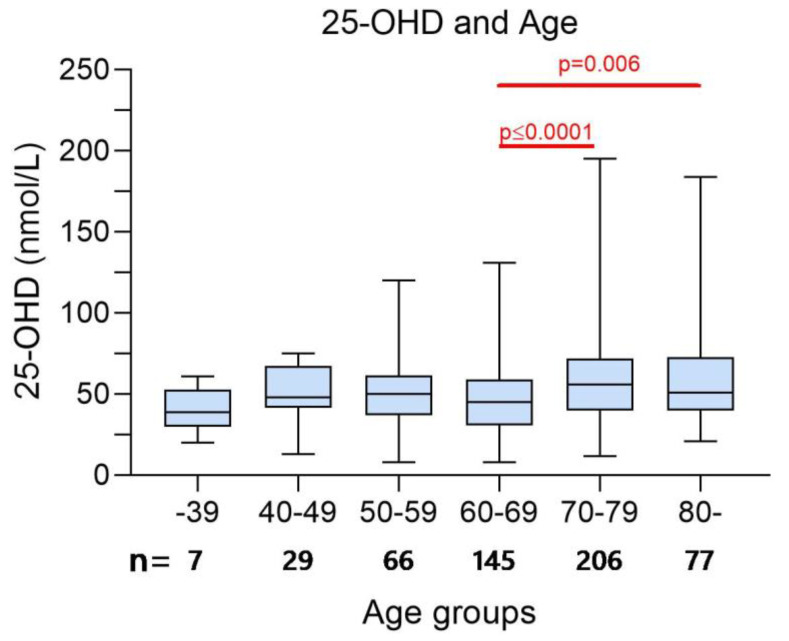
Cross-sectional 25-OHD in relation to age (*n* = 530). Median 25-hydroxyvitamin D (25-OHD) values in nmol/L in the screening cohort (*n* = 530) of the ‘Palliative-D’ study, included the interquartile range and min-max values. Comparisons between groups was performed with Mann–Whitney U. In the 60–69 years age group, the median 25-OHD was lower than in the 70–79 years age group (45 vs. 56 nmol/L, 95% CI −15 to −5, *p* < 0.0001, and in the 80+ years age group (45 vs. 51 nmol/L, 95% CI −15 to −2, *p* = 0.006).

In the screening cohort, median vitamin D levels in patients aged 70–79 years old (*n* = 206) was significantly higher compared to the rest of the screening cohort (56 vs. 51, 95% CI of difference 1–8, *p* = 0.02). In 60–69-year-old patients (*n* = 145), median 25-OHD-levels were instead lower than in other age groups (45 vs. 51 nmol/L, 95% CI of difference 2–10, *p* = 0.005). All other comparisons between age groups and the remaining cohort were not significant. However, when comparing age groups with each other, we also noted that the small group of younger patients (<39 years, *n* = 7) had lower 25-OHD levels than elderly patients. Patients 60–69 years of age had significantly lower 25-OHD levels compared to those who were older, as seen in Figure 4.

### 3.7. 25-OHD in Relation to Survival

#### 3.7.1. Screening Cohort (*n* = 530)

**Figure 5:**
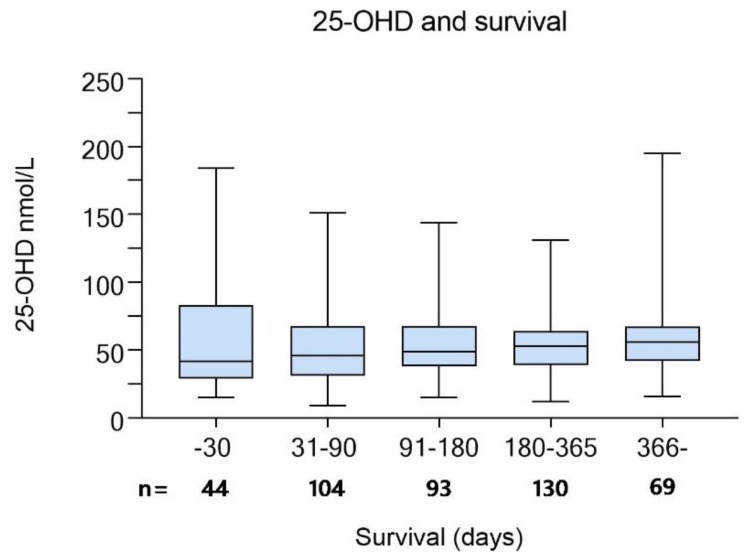
Median 25-hydroxyvitamin D (25-OH) values in nmol/L in deceased patients from the screening cohort (*n* = 440) of the ‘Palliative-D’ study included the interquartile range and min-max values. Comparisons between groups was performed with Mann–Whitney U and there were no statistically significant differences between groups.

**Figure 6:**
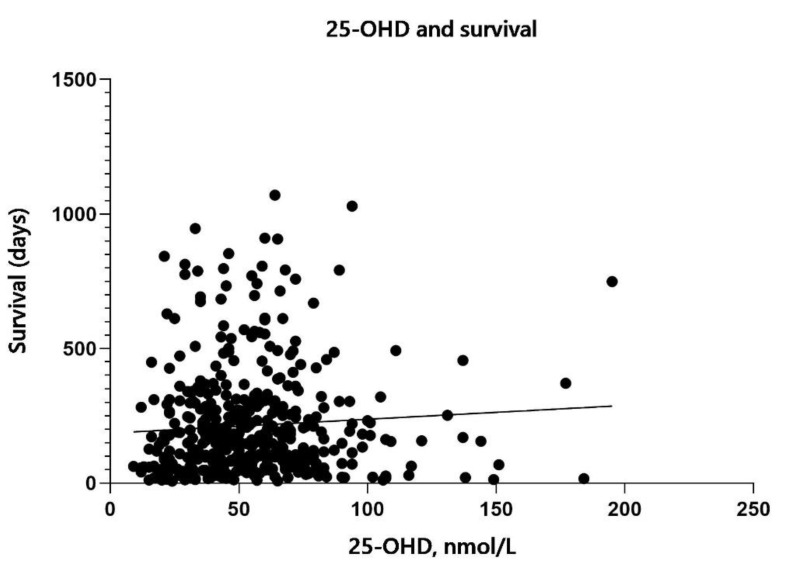
Median 25-hydroxyvitamin D (25-OHD) values in nmol/L in deceased patients from the screening cohort (*n* = 440) of the ‘Palliative-D’ study plotted against survival time in days.

In patients in the screening cohort who were deceased by 9 June 2021 (*n* = 440), 25-OHD at screening did not correlate with survival time (*p* = 0.159). In Figure 5, we present median 25-OHD in patients with a survival time of less than 1 month, 1–3, 3–6, 6–12, and more than 12 months between survival time periods. In Figure 6, we have plotted 25-OHD values versus survival in days. In patients who survived for less than a month after screening (*n* = 44), median 25-OHD was 41.5 and in patients surviving longer than a year (*n* = 59), 56 nmol/L respectively (*p* = 0.11).

#### 3.7.2. Randomized Cohort (*n* = 150)

There was no correlation between 25-OHD and survival after 12 weeks of vitamin D supplementation (*n* = 112, *p* = 0.40).

### 3.8. 25-OHD in Colectomized Patients/Patients with Short Bowel Syndrome

#### 3.8.1. Screening Cohort (*n* = 530)

**Figure 7:**
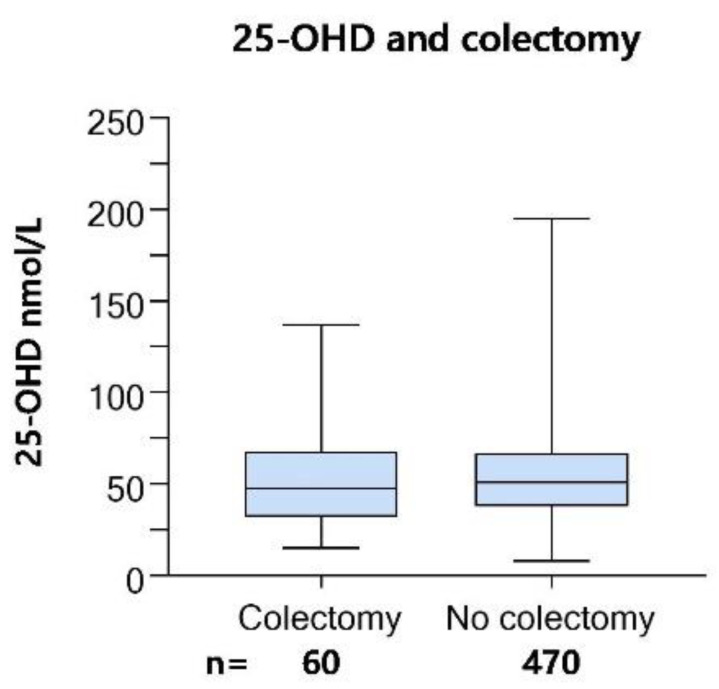
Cross-sectional 25-OHD in colectomized patients. Median 25-hydroxyvitamin D (25-OHD) values in nmol/L in colectomized and non-colectomized patients from the screening cohort of the ‘Palliative-D’-study. Boxes show interquartile range and whiskers min-max values. There was no statistically significant difference between groups (Fischer’s exact test).

There was no difference in median 25-OHD at screening between colectomized patients (*n* = 60) and the rest of the screening cohort (*n* = 470, Figure 7). There were six patients with short bowel syndrome in the screening cohort. In this group, median 25-OHD was 42.5 (range 21–137). Due to the small number of observations, we could not make a comparison of 25-OHD between groups.

#### 3.8.2. Randomized Cohort (*n* = 150)

One patient with short bowel syndrome was supplemented with vitamin D and increased 25-OHD levels from 40 to 98 nmol/L.

## 4. Discussion

In this explorative post-hoc analysis of 25-OHD levels in the ‘Palliative-D’ cohort of severely diseased patients with cancer, 50 percent of patients were vitamin D deficient, and 84% had 25-OHD values below the proposed desired level of 75 nmol/L. Cross-sectional 25-OHD-levels did not vary with time of the year. As shown in the original publication, there was no difference in cross-sectional 25-OHD between men and women, but women had a significantly larger increase in 25-OHD when supplemented with 4000 IU of vitamin D_3_ for 12 weeks. In our material, 60–70-year-old patients had significantly lower 25-OHD levels and 70–80-year-old patients had higher levels compared to other age groups. There was no association between tumor type and 25-OHD. Colectomized patients did not exhibit lower 25-OHD levels than non-colectomized patients. Almost one in four patients supplemented with vitamin D for 12 weeks increased their 25-OHD levels with more than 50 nmol/L. In patients who did not reach vitamin D levels above 50 nmol/L, a majority were male patients with gastrointestinal tumors. There was no association between baseline 25-OHD and survival.

In comparison with other Swedish cohorts with cross-sectional data on 25-OHD, our cohort of severely diseased patients had higher 25-OHD values compared to comparatively older Swedish nursing home residents [34,39,51], however values were lower than in healthy elderly [39]. We also observed significantly less seasonal variation in 25-OHD levels compared to healthy (and younger) Swedish cohorts [29,31]. We suggest that as patients with advanced cancer spend less time outside, consequently their 25-OHD levels rely less on sun exposure.

As seen in Table 1, 25-OHD levels in palliative cohorts with mixed tumor types vary greatly [40,41,42,43,46,48,49]. Our cohort is the yet largest to be studied. Patients screened in ‘Palliative-D’ had higher 25-OHD levels compared to recently studied cohorts in Spain and Turkey [24,40], well in line with Australian and US experiences [43,45,46,48,49]. The fact that 25-OHD-levels did not differ between tumor types is consistent with previous findings [45].

In our material, women increased their 25-OHD-levels more than men when supplemented with vitamin D, and more specifically a small number of women had very large increases in 25-OHD. We do not know whether this is due to sex differences in vitamin D uptake and metabolization, whether these individuals took more vitamin D supplementation than prescribed in the study, or otherwise changed their lifestyle and eating habits to increase 25-OHD.

In the multivariate analysis of ‘Palliative-D’, colectomy and cancer type did not affect results regarding pain, infections, and quality of life [47]. However, all vitamin D supplemented patients with very small changes in 25-OHD after 12 weeks had GI-tumors. This indicates that at least in some GI-cancer patients, reduced uptake of vitamin D may be an issue, as seen in other studies on patients with GI cancer [52].

We consider the fact that vitamin D supplementation is recommended in all citizens aged 75 years and above in Sweden plays a role in the higher levels of 25-OHD observed in the elderly in our cohort [2]. In the small group of young patients, 25-OHD levels were low. We do not interpret this as a difference between groups attributable to age itself. Rather, there were individuals in this group with very long disease trajectories and many lines of palliative oncological treatment.

We noted that 10 patients had very high levels of 25-OHD at screening, although ongoing vitamin D supplementation was an exclusion criterion in the ‘Palliative-D’ study. These patients were recruited to the study during all four quarters of the year. To us it seems unlikely that levels above 150 nmol/L are attained solely through sun exposure, and we suspect that these patients were taking vitamin D supplementation. Use of vitamin D supplementation is presently very common in cancer patients [26]. In Sweden, we do not routinely screen for vitamin D deficiency, and possibly some patients enrolled in the study mainly to have their vitamin D status checked.

Several patients had high 25-OHD levels at screening, and a few also reached levels above 150 nmol/L after 12 weeks of supplementation. The safety, over time, of such high levels is debated [8,53].

A strength of this study is the size of the screening cohort and the fact that we included many different types of cancer types. Limitations include the fact that the subgroups are small, especially when analyzing patients followed for 12 weeks, and this makes comparisons across subgroups less reliable. Furthermore, only 10% of patients were recruited to the screening cohort during the months of June, July, and August. We did not collect data on dietary intake and did not assess levels of the parathyroid hormone (PTH). However, dietary intake could not account for the large increases of 25-OHD in supplemented patients.

## 5. Conclusions

Levels of 25-OHD in palliative cancer patients in northern latitudes have less seasonal variation than healthy populations from the same latitudes. The type of cancer does not predict vitamin D levels in a palliative setting. There is large inter-individual variation in cross-sectional 25-OHD levels, indicating that some patients take larger doses of vitamin D supplementation. Even in a severely diseased population, patients respond well to vitamin D supplementation with adequate increase in 25-OHD levels, regardless of season, age, tumor type, or colectomy, however the increase may be more pronounced in women than in men.

## Data Availability

Raw data from the “Palliative-D” study is available from the corresponding author upon request.

## Supplementary Materials

The following are available online at https://www.mdpi.com/article/10.3390/nu14030602/s1, Table S1: Cross sectional cohorts with measurements of 25-OHD in Sweden, Table S2: Baseline levels and change in 25-OHD (nmol/L) after 12 weeks of supplementation of vitamin D3 4000 IU/day in relation to cutoff levels in a cohort of patients with cancer in palliative phase, Table S3: 25-OHD in the screening cohort (*n* = 530) in relation to season, Table S4: Change in 25-OHD over 12 weeks in relation to season in vitamin D supplemented patients and in patients receiving placebo.
