# Supplementary material for: 25-Hydroxyvitamin D in Cancer Patients Admitted to Palliative Care: A Post-Hoc Analysis of the Swedish Trial ‘Palliative-D’
Source: Nutrients. 2022 Jan 29;14(3):602. doi: 10.3390/nu14030602 (PMC8840761; doi:10.3390/nu14030602)
Supplement: Supplementary file 1 [file nutrients-14-00602-s001.zip › nutrients-1526687-supplementary.pdf]

Table S1. Cross sectional cohorts with measurements of 25-OHD in Sweden

Author, Study	Cohort, Location Time Period	Study population, Sex, age	25-OHD (nmol/L)	Seasonal variation in 25-OHD (nmol/L)	Proportions of vit D deficient participants (25-OHD, nmol/L)	Comments
**Arnljots 2017 [1]**	Nursing homes, 57-58°N, Jan-March 2012	n=545, 32% men<br>Mean age 86	27 (4–125)	No data	41% < 25<br>82% < 50	17% on vitamin D supplementation
**Samefors 2014 [2] SHADES**	Nursing homes Southern Sweden, 2007-11	n=333, 32% men<br>Mean age women 86, men 83	40 (16)<br>Min-max 12–120	May-Oct 39<br>Nov-April 35<br>(mean, p<0.01)	80% < 50	Vitamin D supplementation = exclusion criterion
**Samefors 2017 [3] CARDIPP**	Community dwelling, DM II, 57-58°N, 2005-08	n=698, 66% men<br>Mean age 60	51 (22)	Correction for season in multivariate analysis	8% < 25<br>55% < 50	Vitamin D supplementation = exclusion criterion
**Carlsson 2016 [4]**	County hospital + general public 56,7°N Feb 2014-April -15	n=475<br>45% men<br>Mean age 78 in healthy controls	HC ≥75 years: 74 (22-154)<br>Fractures: 50 (10-128)<br>Stroke: 62 (16-135)<br>FA: 56 (16-165)<br>Nursing homes: 38 (15-132)	Small difference April-Sept vs Oct-March (p=0.003).	Healthy controls:<br>13% < 50<br>Nursing home residents:<br>75% < 50	Vitamin D prescription: 15.5% FA, 0% HC. No information on supplements.
**Klingberg 2015 [5]**	Blood + trc donors 57.4°N April-Nov 2008	n=540 blood donors, 62% men, mean age 40.5<br>n=75 trc donors, 92% men, mean age 46	Mean 60 (10-224)	July: 82 (26) vs<br>Feb: 48 (21), p<0.001<br>Q1: 48 (20) vs<br>Q3: 78 (27), p<0.001	Q1: 59% <50, 88% <75<br>Q2: 36% <50, 76% <75<br>Q3: 8% <50, 45% <75<br>Q4: 30% <50, 80% <75	8 % on multivitamin supplementation
**Nälsén 2020 [6] Riksmaten**	All Swedish regions 2010-11	n=268	May-Oct 65 (24-168)<br>Nov-Apr 55 (20-94)	May-Oct:<br>5%<30, 18%<50<br>Nov-Apr:<br>x%<30, 40%<50	See "seasonal variation"	7.5% on dietary supplements
**Buchebner 2019 [7] OPRA**	Community dwelling, Southern Sweden, 1995-99, 16 years follow up	75 years: n=1044<br>80 years n=715<br>85 years n=382<br>100% women	75 years: 62 (19)<br>80 years: 78 (30)<br>85 years: 79 (26)	No data	Data not shown	75 years:<br>6 % vit D suppl.<br>80 years: 47 %<br>85 years: 52%
**Lundström 2019 [8]**	Overweight, impaired glucose tolerance 59°N, 2006-08	n=158<br>45% men	Before summer: 55 (22)<br>After summer: 66 (21)	Seasonal variative also in<br>non-users (p 0.03)	Before summer:<br>37% < 30 ,<br>After summer:<br>4% < 30, 18% < 50	13 % on supplements, mean dose 7.5 ug
**Björk 2019[9]**	Community dwelling (MrOS Sweden), 2000-02	n=2924<br>100% Men<br>Mean age 75	79 (22.5)	Vitamin D values were deseasonalized	1% < 25	No information on vitamin D ¨ supplementation

For cross-sectional 25-OHD, median values with (min-max) or mean values with standard deviation (SD) are presented**. Abbreviations:** 25-OHD: 25-hydroxyvitamin D, DM: Diabetes mellitus, FA: Frequent admissions, HC: healthy controls °N: degrees North (latitude), Q: Quarter (of the year), trc: thrombocytes, vit:vitamin

References

1. Arnljots, R.; Thorn, J.; Elm, M.; Moore, M.; Sundvall, P.D. Vitamin D deficiency was common among nursing home residents and associated with dementia: a cross sectional study of 545 Swedish nursing home residents. *BMC Geriatr* **2017**, *17*, 229.
2. Samefors, M.; Östgren, C.J.; Mölstad, S.; Lannering, C.; Midlöv, P.; Tengblad, A. Vitamin D deficiency in elderly people in Swedish nursing homes is associated with increased mortality. *Eur J Endocrinol* **2014**, *170*, 667-675.
3. Samefors, M.; Scragg, R.; Länne, T.; Nyström, F.H.; Östgren, C.J. Association between serum 25(OH)D(3) and cardiovascular morbidity and mortality in people with Type 2 diabetes: a community-based cohort study. *Diabet Med* **2017**, *34*, 372-379.
4. Carlsson, M.; Wanby, P.; Brudin, L.; Lexne, E.; Mathold, K.; Nobin, R.; Ericson, L.; Nordqvist, O.; Petersson, G. Older Swedish Adults with High Self-Perceived Health Show Optimal 25-Hydroxyvitamin D Levels Whereas Vitamin D Status Is Low in Patients with High Disease Burden. *Nutrients* **2016**, *8*.
5. Klingberg, E.; Oleröd, G.; Konar, J.; Petzold, M.; Hammarsten, O. Seasonal variations in serum 25-hydroxy vitamin D levels in a Swedish cohort. *Endocrine* **2015**, *49*, 800-808.
6. Nälsén, C.; Becker, W.; Pearson, M.; Ridefelt, P.; Lindroos, A.K.; Kotova, N.; Mattisson, I. Vitamin D status in children and adults in Sweden: dietary intake and 25-hydroxyvitamin D concentrations in children aged 10-12 years and adults aged 18-80 years. *J Nutr Sci* **2020**, *9*, e47.
7. Buchebner, D.; Bartosch, P.; Malmgren, L.; McGuigan, F.E.; Gerdhem, P.; Akesson, K.E. Association Between Vitamin D, Frailty, and Progression of Frailty in Community-Dwelling Older Women. *J Clin Endocrinol Metab* **2019**, *104*, 6139-6147.
8. Lundström, P.; Caidahl, K.; Eriksson, M.J.; Fritz, T.; Krook, A.; Zierath, J.R.; Rickenlund, A. Changes in Vitamin D Status in Overweight Middle-Aged Adults with or without Impaired Glucose Metabolism in Two Consecutive Nordic Summers. *J Nutr Metab* **2019**, *2019*, 1840374.
9. Björk, A.; Ribom, E.; Johansson, G.; Scragg, R.; Mellström, D.; Grundberg, E.; Ohlsson, C.; Karlsson, M.; Ljunggren, Ö.; Kindmark, A. Variations in the vitamin D receptor gene are not associated with measures of muscle strength, physical performance, or falls in elderly men. Data from MrOS Sweden. *J Steroid Biochem Mol Biol* **2019**, *187*, 160-165.

Table S2. Baseline levels and change in 25-OHD (nmol/L) after 12 weeks supplementation of vitamin D3 4000 IU/day in relation to cutoff levels in a cohort of patients with cancer in palliative phase.

Baseline 25-OHD	Numper of patients (%)	25-OHD at 12 weeks				
		≤ 25	26-50	51-75	76-100	>100
**≤ 25**	9 (13)	0	2 (22)	3 (33)	2 (22)	2 (22)
**26-50**	58 (87)	0	7 (12)	15 (26)	23 (40)	13 (22)

Table S3. 25-OHD in the screening cohort (n=530) i relation to season.

**Time period**	**Number of cases,** N (%)	**25-OHD Median (IQR)** ***Min-max***	**25-OHD Mean (SD)**	**25-OHD <25 n (%)**	**25-OHD 25-49 n (%)**	**25-OHD 50-74 n (%)**	**25-OHD 75-124 n (%)**	**25-OHD ≥125 n (%)**	Comparison of mean 25-OHD-levels between Q1-Q4
**Q1**	186<br>*Jan: 67*<br>*Feb: 51*<br>*March: 68*	52<br>(37-67)<br>*8-195*	55,4<br>(28.4)	18 (9)	69 (37)	72 (39)	22 (12)	5 (3)	Q1vs Q2: 0.85<br>Q1vs Q3: 0.86<br>Q1vs Q4: 0.86
**Q2**	85<br>*April: 33*<br>*May: 40*<br>*June: 12*	50<br>(35-72)<br>*15-149*	56.1<br>(28.4)	10 (12)	32 (38)	24 (28)	16 (19)	3 (3)	Q2 vs Q3: 0.74<br>Q2 vs Q4: 0.25
**Q3**	84<br>*July: 16*<br>*August: 24*<br>*Sept: 44*	53<br>(39-70)<br>*8-129*	54.7<br>(23.9)	8 (10)	27 (32)	33 (39)	15 (18)	1 (1)	Q3 vs Q4: 0.44
**Q4**	175<br>Oct: 34<br>Nov: 92<br>Dec: 49	50<br>(39-63)<br>*9-175*	52.3<br>(22.9)	18 (10)	67 (38)	64 (37)	25 (14)	1 (1)	
**Nov-April**	360	50<br>(37-66)<br>*8-195*	54.2<br>(26.5)	35 (10)	140 (39)	130 (36)	48 (13)	7 (2)	Nov-April vs May-Oct: 0.82
**May-Oct**	170	53<br>38-70<br>*8-149*	54.8<br>(24.8)	19 (11)	55 (32)	63 (37)	30 (18)	3 (2)	
**All**	530	ref	ref	54 (10)	210 (40)	183 (34)	73 (14)	10 (2)	

Table S4. Change in 25-OHD over 12 weeks in relation to season in vitamin D supplemented patients and in patients receiving placebo.

**Change in 25-OHD in nmol/L over 12 weeks in patients randomized to placebo**			
Time period	Number of subjects	Change in 25-OHD<br>**Median** (IQR) *min-max*	p=
Q1	21	**3** (-3 – 14)<br>*-25 – 44*	Q1 vs Q2: 0.90<br>Q1 vs Q3: 0.15<br>Q1 vs Q4: 0.03
Q2	14	**4** (-6 – 13.5),<br>*-15 – 23*	Q2 vs Q3: 0.37<br>Q2 vs Q4: 0.15
Q3	12	**0** (-7.25– 5.5)<br>*-10 – 13*	Q3 vs Q4:0.34
Q4	31	**-3** (-10 – 6.5)<br>*-17– 20*	
May-October	24	**0** (-6 – 12)<br>*-25 – 44*	Nov-April vs May-Oct: 0.60
Nov-April	59	**1** (-8 – 6)<br>*-13 – 23*	
All	83	**0** (-6 – 10.5)<br>*-25 – 44*	
**Change in 25-OHD over 12 weeks in patients randomized to vitamin D 4000 IU/day**			
Time period	Number of subjects	Change in 25OHD<br>**Median** (IQR) *min-max*	p=
Q1	19	**44** (36 – 62)<br>*26 – 116*	Q1vsQ2: 0.87<br>Q1vsQ30.27<br>Q1vsQ4: 0.07
Q2	11	**47** (35 – 65)<br>*7– 84*	Q2vsQ4: 0.30<br>Q2 vs Q3: 0.54
Q3	12	**35.5** (8.5 – 61)<br>*-2 – 122*	Q3 vs Q4: 0.98
Q4	25	**37** (20 – 55)<br>*-2 – 94*	
May-October	24	**41** (20 – 61),<br>*-2 – 122*	Nov-April vs May-Oct: 0.95
Nov-April	43	**39.5** (28.5 – 55)<br>*-2 - 116*	
All	67	**39.5** (27 – 58.5)<br>*-2 – 122*	0.95
